# Uneven malaria transmission in geographically distinct districts of Bobo-Dioulasso, Burkina Faso

**DOI:** 10.1186/s13071-018-2857-x

**Published:** 2018-05-11

**Authors:** Dieudonné Diloma Soma, Daouda Kassié, Seydou Sanou, Fatou Biribama Karama, Ali Ouari, Wadaka Mamai, Georges Anicet Ouédraogo, Gérard Salem, Roch Kounbobr Dabiré, Florence Fournet

**Affiliations:** 1 Institut de Recherche en Sciences de la Santé/Centre Muraz, Bobo-Dioulasso, BP 545 Burkina Faso; 2 Université Nazi Boni, Bobo-Dioulasso, BP 109 Burkina Faso; 3 Université Paris Ouest Nanterre La Défense, 200 Avenue de la République, 92000 Nanterre, France; 4 UMR MIVEGEC (IRD, CNRS, UM), Institut de Recherche pour le Développement, 911, Avenue Agropolis, BP 64501, 34394 Montpellier Cedex 5, France; 5 UMR ASTRE, CIRAD, CIRAD TA C-22/E, Campus International de Baillarguet, 34398 Montpellier Cedex 5, France; 6 Insect Pest Control Laboratory, Joint FAO/IAEA Division of Nuclear Techniques in Food and Agriculture, Vienna, Austria; 7 Institut de Recherche Agricole pour le Développement (IRAD), Yaoundé, Cameroon; 8 CEPED, Institut de Recherche pour le Développement, 45 rue des Saints-Pères, 75006 Paris, France

**Keywords:** Urbanization, Spatial heterogeneity, Malaria, *Anopheles* bites exposure, Burkina Faso

## Abstract

**Background:**

Urbanization is a main trend in developing countries and leads to health transition. Although non-communicable diseases are increasing in cities of low-income countries, vector-borne diseases such as malaria, are still present. In the case of malaria, transmission is lower than in rural areas, but is uneven and not well documented. In this study, we wanted to evaluate intra-urban malaria transmission in a West African country (Burkina Faso).

**Methods:**

A cross-sectional study on 847 adults (35 to 59 year-old) and 881 children (6 months to 5 year-old) living in 1045 households of four districts (Dogona, Yeguere, Tounouma and Secteur 25) of Bobo-Dioulasso was performed between October and November 2013. The districts were selected according to a geographical approach that took into account the city heterogeneity. Malaria prevalence was evaluated using thick and thin blood smears. Human exposure to *Anopheles* bites was measured by assessing the level of IgG against the *Anopheles* gSG6-P1 salivary peptide. Adult mosquitoes were collected using CDC traps and indoor insecticide spraying in some houses of the four neighbourhoods. The *Anopheles* species and *Plasmodium falciparum* infection rate were determined using PCR assays.

**Results:**

In this study, 98.5% of the malaria infections were due to *Plasmodium falciparum*. Malaria transmission occurred in the four districts. Malaria prevalence was higher in children than in adults (19.2 *vs* 4.4%), and higher in the central districts than in the peripheral ones (*P* = 0.001). The median IgG level was more elevated in *P. falciparum-*infected than in non-infected individuals (*P* < 0.001). *Anopheles arabiensis* was the main vector identified (83.2%; 227 of the 273 tested mosquito specimens). Five *P. falciparum*-infected mosquitoes were caught, and they were all caught in the central district of Tounouma where 28.6% (14/49) of the tested blood-fed mosquito specimens had a human blood meal.

**Conclusions:**

This study showed that urban malaria transmission occurred in Bobo-Dioulasso, in all the four studied areas, but mainly in central districts. Environmental determinants primarily explain this situation, which calls for better urban management.

## Background

Vector-borne diseases continue to be a threat in most low-income countries, particularly malaria [1]. About 3.2 billion people, almost half of the world population, are at risk of malaria that caused an estimated 216 million cases and about 445 000 deaths in 2016 [1]. *Plasmodium falciparum* is responsible for most (99%) of these deaths [1] in Africa. Specifically, 90% of malaria cases and 91% of malaria deaths occurred in sub-Saharan Africa in 2016 [1]. In Burkina Faso, malaria represents the most common cause of medical consultation, with 7 million cases and 5,379 deaths in 2015 [1]. Moreover, due to the lack of good access to healthcare, the situation in the field may be more alarming.

Urbanization is accelerating in Africa, and demographic projections suggest that this phenomenon will further increase in the future [2]. It is generally thought that due to the higher concentration of healthcare facilities, urban populations enjoy better health than rural populations. Indeed, different studies showed that malaria transmission is lower in urban than in rural areas because of the unsuitability of urban environments for its vectors [3]. However, malaria epidemiology is different in urban contexts, and the delayed acquisition of a protective immunity causes more severe malaria [4]. Some studies have demonstrated that *Anopheles* vectors can adapt to the urban environment. For instance, urban agriculture offers great conditions for the development of anopheline larvae, as observed in small towns, such as Bouaké in Ivory Coast [5], but also in capital cities, such as Dakar in Senegal [6] and Antananarivo in Madagascar [7]. Human activities (for instance, brick making pits) also create breeding sites for *Anopheles* larvae [8, 9].

Moreover, urbanization is unequal in time and space, and also between continents, countries and within cities [10]. This urban heterogeneity could influence the effectiveness of health policies, particularly in the case of malaria, calling for a better assessment of local situations. The objectives of this study were to assess and compare the spatial patterns of malaria transmission within Bobo-Dioulasso (Burkina Faso) to optimize the environmental management and improve urban health.

## Methods

### Study site and design

Bobo-Dioulasso is the second largest city in Burkina Faso with an estimated population of 813,810 inhabitants in 2012 [11]. It is located in the southwestern part of the country (11°10'7.31"N, 4°17'52.24"W). Malaria is present throughout the year with a peak of transmission during the rainy season, between May and October. *Plasmodium s*pecies, mainly *P. falciparum* are transmitted by *Anopheles gambiae s.s*. [1, 12, 13].

**Fig. 1:**
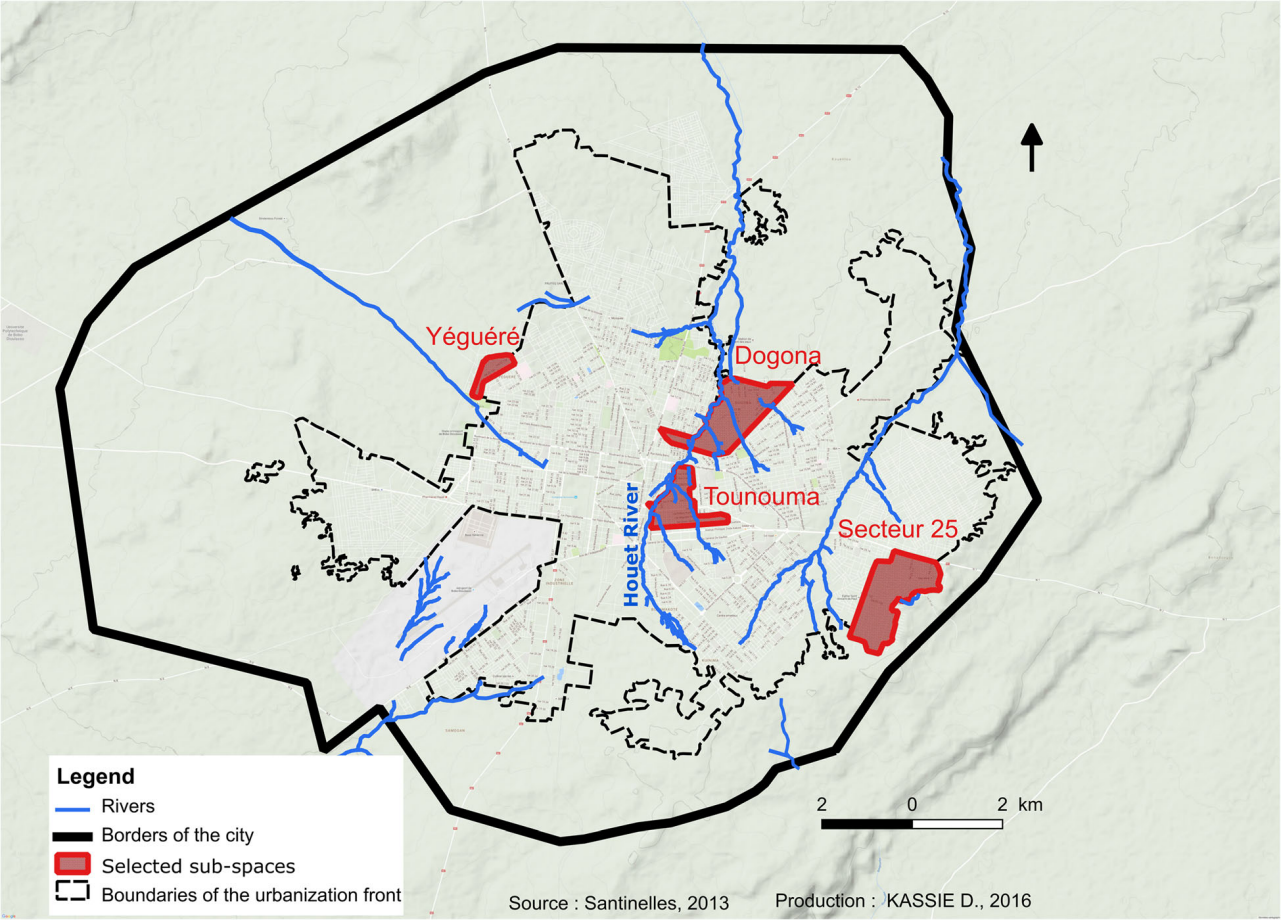
Localization of the four districts and hydrographic network

The study was conducted in November 2013 in four districts: Dogona, Yeguere, Tounouma and Secteur 25 (Fig. 1). These districts were selected using a geographical approach that took into account the city heterogeneity [14]. This approach was based on the analysis of the built environment and the distribution of infrastructures, such as healthcare facilities and public fountains, using aerial photographs and satellite images. Tounouma is an old urbanized district in the central part of the town (Table 1). Dogona is located close to the city centre and has been more recently urbanized. These districts are crossed by the Houet River, a permanent river whose banks are heavily exploited by market gardening. Conversely, Secteur 25 is a peripheral, recently urbanized area that is still under construction and where healthcare facilities are scarce, different from Dogona and Tounouma. Yeguere is a peripheral, spontaneously formed district that completely lacks urban equipment. In Dogona and Tounouma, water is mainly provided by public fountains. In Secteur 25 individual connections are preferred.

**Table 1:** Presentation of the four districts according to the urbanization, position in the city, access to water supply and health care

Districts	Age of district (years)	Location	Water supply	Access to health care	Distance from hydrographic network^a^ (m)
Yeguere	26	Peripheral	None	Bad	815
Secteur 25	17	Peripheral	Individual	Scarce	597
Tounouma	> 55	Central	Public fountain	Good	152
Dogona	51	Central	Public fountain	Good	168

^a^Average distance between households surveyed and hydropgraphic network

The surveyed households were randomly selected on a cadastral map using the sampling tool of the ArcGis10 software. Distance from hydrographic network was calculated for each household and then for the district (Table 1). In each included household, one adult between 35 and 59 years of age and one child between 5 and 59 months of age were randomly selected. During household visits, information about their habits, particularly number of nets per household, date of acquisition, and use of mosquito net the night before the survey, was collected.

### Malaria parasite detection

Blood samples were collected by finger pricking to prepare thick and thin blood smears. Axillary temperature was measured with an electronic thermometer after basic clinical examination. If the temperature was higher than 37.5 °C, a malaria rapid diagnostic test (mRDT) was performed according to the manufacturer’s instructions. People with a positive mRDT were immediately treated for free, as recommended by the National Malaria Control Programme of Burkina Faso.

Blood smears were fixed and stained with 10% Giemsa and examined at the Institut de Recherche en Sciences de la Santé (IRSS) in Bobo-Dioulasso to identify *Plasmodium* species. *Plasmodium falciparum* parasites (asexual stages) were counted in 200 leukocytes and parasite density was calculated for a fixed leukocyte count of 8000 cells/μl of blood. All thick and thin blood smears were read independently by two skilled technicians. Comparison of their results indicated that the estimated rates of parasite detection and parasite density did not differ significantly between technicians. Quality control was regularly performed on randomly selected samples, representing 10% of all thick smears. A malaria case was defined as the presence of fever or history of fever during the past 48 h and parasitaemia > 0.

### Exposure to *Anopheles* bites

Blood samples for assessing mosquito bite exposure were collected on filter paper (Whatman™ 3MM Chr, Paris, France). They were stored at 4 °C until use. The IgG response to the gSG6-P1 salivary peptide, which is specific for *An. gambiae*, was evaluated using enzyme-linked immunosorbent assay (ELISA), as previously described [15, 16]. Optical density (OD) was measured according to the protocol described by Poinsignon et al. [15]. The IgG response to the gSG6-P1 peptide was expressed as the ∆OD value, as described by Sagna et al. [17]. The average specific immune response threshold of non-*Anopheles* exposed individuals (ΔODneg) was: ΔODneg ± 3 standard deviations = 0.204. An individual was considered to be an immune responder when ∆OD > 0.204.

### Entomological survey

Mosquito collections were carried out inside and just outside four houses in each of the four districts using CDC traps during two consecutive nights (between 20:00 and 06:00 h). Indoor resting females were caught by spraying four other houses close to the CDC-sampling houses with insecticide aerosols between 06:00 and 09:00 h during the same two consecutive days. Dead or knocked-down mosquitoes were collected on white sheets laid down on the floor of the sprayed houses. Species identification was performed for *An. gambiae* complex specimens using the PCR method described by Santolamazza et al. [18]. Their infection rate was evaluated by PCR amplification as described by Morlais et al. [19]. The blood meal origin was determined by ELISA in blood-fed females [20] using antibodies against human and most frequently found animals in the study sites: cattle, sheep, dogs, donkeys and pigs.

### Statistical analysis

The number of households to be surveyed and then the number of participants were calculated in order to observe significant differences between the four districts at the 5% threshold with a precision of 2.6%, based on a prevalence of 5% (the smallest expected prevalence for malaria in adults). Malaria prevalence was calculated as the number of malaria cases reported to the population considered (children, adults). Malaria prevalence in the four districts as well as in the central and peripheral areas was compared by a Chi-square test. Parasitemia was calculated among those that had a detectable parasitemia. Variance analysis allowed the comparison between districts. The immunological assays were performed on a sub-sample (*n* = 273) of children and adults randomly selected from the whole sample. Normality distribution was tested using the Kolmogorov-Smirnov and Shapiro-Wilk tests. The non-parametric Mann-Whitney test was used to compare IgG Ab levels between independent groups, and the Kruskal-Wallis test for comparisons of more than two groups. IgG antibody levels between districts were analyzed using analysis of variance (ANOVA) with Tukey’s honestly significant difference (HSD) post-hoc tests. A *P*-value < 0.05 was considered as significant. Graph Pad Prism 5.0 Software and Microsoft Excel 2013 (Microsoft®, New York, USA) were used to produce graphics and perform statistical analyses.

## Results

### Prevalence of malaria infection

This study included 1728 individuals (847 adults and 881 children) living in 1045 households (Table 2). Long-lasting insecticidal nets (LLIN) were distributed in July 2013 during the national campaign, just before the survey during which 97.6% of adults and 91% of children declared to have slept under their LLIN the night before.

**Table 2:** Characteristics of malaria in the different districts of Bobo-Dioulasso, 2013

District	Households	Children					Adults				
		*n*	Malaria cases	%	95% CI	Average parasitemia/μl^a^	*n*	Malaria cases	%	95% CI	Average parasitemia/μl^a^
Yeguere	234	208	36	17.3	9.9–21.1	4869	199	5	2.5	0–4.1	118
Secteur 25	290	223	34	15.2	8.2–18.8	1584	215	7	3.3	0–5.1	68
Tounouma	286	248	46	18.5	10.9–22.4	4346	240	13	5.4	1.0–7.7	546
Dogona	235	202	53	26.2	17.6–30.6	4782	193	12	6.2	1.5–8.6	310
All districts	1045	881	169	19.2	11.5–23.1	3872	847	37	4.4	0.4–6.5	275

^a^Among individuals with detectable parasitemia *Abbreviation*: CI, confidence interval

A total of 206 cases of malaria were reported (Table 2). The prevalence of malaria infection was 12% (206/1728) in the whole population and was higher in children (19.2%, 169/881; 95% CI: 11.5–23.1%) than in adults (4.4%, 37/847; 95% CI: 1.5–8.6%) (Table 2). More malaria cases were observed in Dogona (65/206) and Tounouna (59/206). In Secteur 25 and Yeguere 41 malaria cases were registered in each district. Comparative analyses that took into account the intra-urban distribution showed that malaria prevalence in children was significantly different in the four districts (*P* = 0.026). No difference in malaria prevalence distribution among districts was observed in adults. Comparing neighborhoods to two showed that Dogona was significantly different from Secteur 25 and Yeguere, but quite similar to Tounouma (Table 3). Comparison of the malaria prevalence for the whole population (children and adults) between central (Tounouma and Dogona) and peripheral districts (Yeguere and Secteur 25) showed that malaria prevalence was significantly higher in the central districts than in the peripheral ones (*P* = 0.001). *Plasmodium falciparum* was identified in 98.5% (205/208) and *P. malariae* in 1.4% (3/208) of all infected individuals in the four districts. The average parasitaemia was 3872 and 275 *P. falciparum* asexual parasites/μl of blood in children and adults, respectively (Table 2). Differences in parasitaemia between districts (higher in Tounouma and lower in Secteur 25) were significant only for adults (*P* = 0.019).

**Table 3:** Pairwise comparisons of the districts (probability after Chi-square test)

	Secteur 25		Tounouma		Dogona	
	Chi-square	*P*-value	Chi-square	*P*-value	Chi-square	*P*-value
Yeguere	0.336	0.562	0.118	0.731	4.808	0.028
Secteur 25	-	-	0.908	0.223	7.864	0.004
Tounouma	-	-	-	-	3.835	0.048
Dogona	-	-	-	-	-	-

### IgG against the *Anopheles* gSG6-P1 salivary peptide

**Fig. 2:**
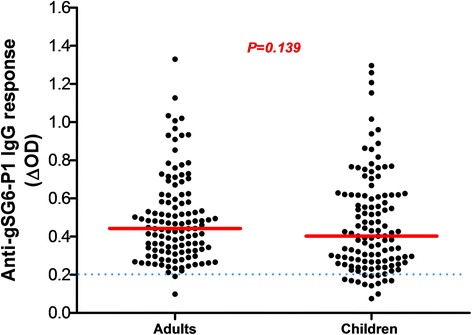
Anti-gSG6-P1 IgG level in the four districts. Red bars indicate the median value in each group and the horizontal blue dotted line represents the cut-off for immune responders (∆OD > 0.204). Data were compared with the Kruskal-Wallis test. *Abbreviation*: *n*, number of samples

**Fig. 3:**
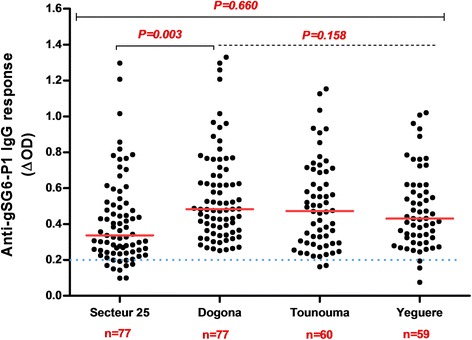
Anti-gSG6-P1 IgG response according to the district geographic position. Red bars indicate the median value in each group and the horizontal blue dotted line represents the cut-off for immune responders (∆OD > 0.204). Peripheral districts, Secteur 25 and Yeguere; central districts, Tounouma, Dogona. Data were compared with the Mann-Whitney test. *Abbreviation*: *n*, number of samples

**Fig. 4:**
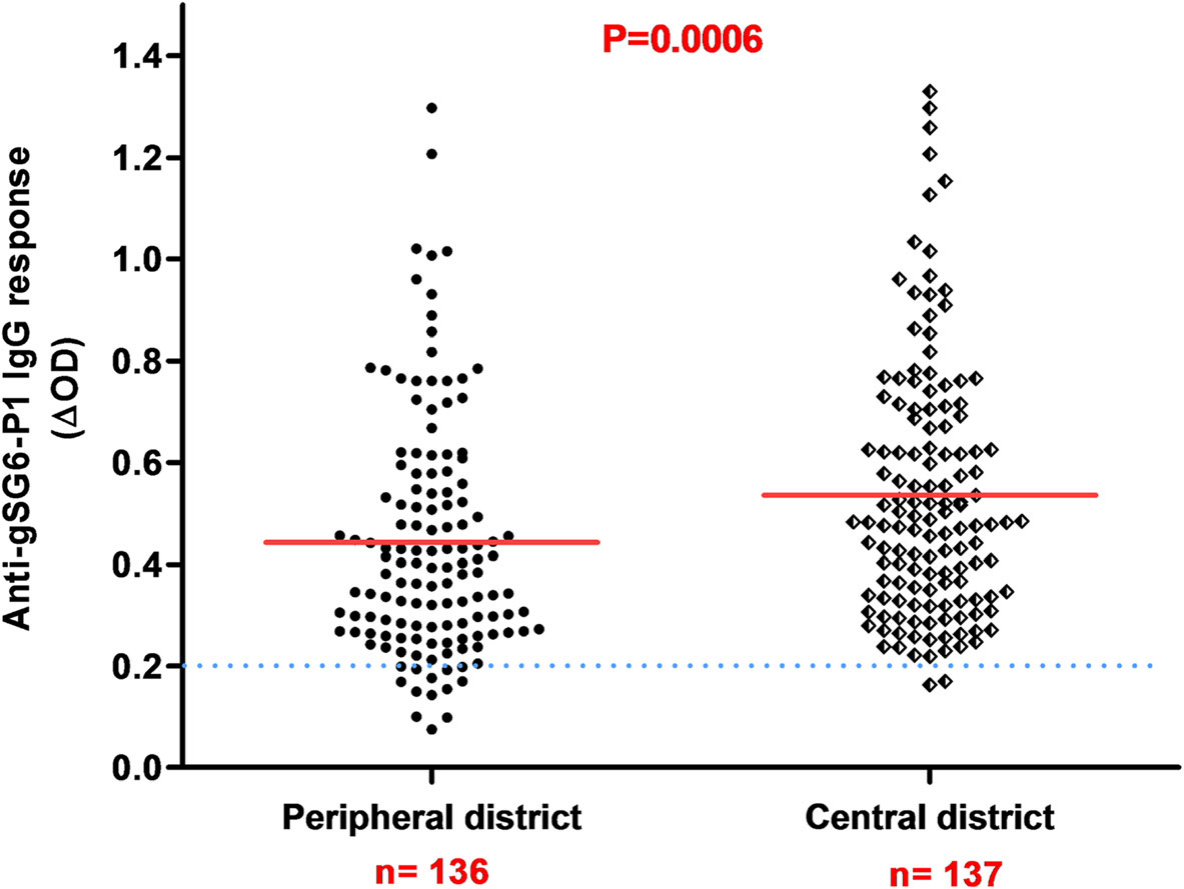
Anti-gSG6-P1 IgG levels in children and adults according to their malaria infection status. Red bars indicate the median value in each group and the horizontal blue dotted line represents the cut-off for immune responders (∆OD > 0.204). Data were compared with the Mann-Whitney test. *Abbreviations*: Uninf-Adults, non-infected adults; Inf-Adults, infected adults; Uninf-Child, non-infected children; Inf-Child, infected children; n, number of samples

From all samples (*n* = 1728), a subsample of 273 blood spots was randomly selected to evaluate the exposure to *Anopheles* bites by assessing the immune response to the salivary gSG6-P1 peptide. The proportion of children and adults tested positive for an IgG response (above the threshold) was 89% (122/137) and 99% (135/137), respectively. No significant difference in the serology analysis based on age was observed among adults (ANOVA: *F*_(4, 112)_ = 1.980, *P*= 0.1023) and children (ANOVA: *F*_(4, 115)_ = 2.365, *P* = 0.0570). No significant difference was observed between adults and children, (Mann-Whitney U-test: *U* = 6295, *n*_1_ = 118, *n*_2_ = 120, *P* = 0.1396) (Fig. 2). Analysis according to the district showed that the anti-gSG6-P1 IgG levels were significantly lower in Secteur 25 than in Tounouma, Dogona and Yeguere (Mann-Whitney U-test, *U *= 1781, *n*_1_ = 77, *n*_2_ = 77, *P* = 0.0003) where the anti-gSG6-P1 IgG responses were comparable (Kruskal-Wallis H-test: *χ*^2^ = 3.54, *df* = 2, *P* = 0.158) (Fig. 3). Analysis according to the district geographical position (central *vs* peripheral) showed that the anti-gSG6 P1 IgG response was significantly higher in the central than in the peripheral districts (Mann-Whitney test, *U *= 7072, *n*_1_ = 136, *n*_2_ = 137, *P* = 0.0006) (Fig. 4).

**Fig. 5:**
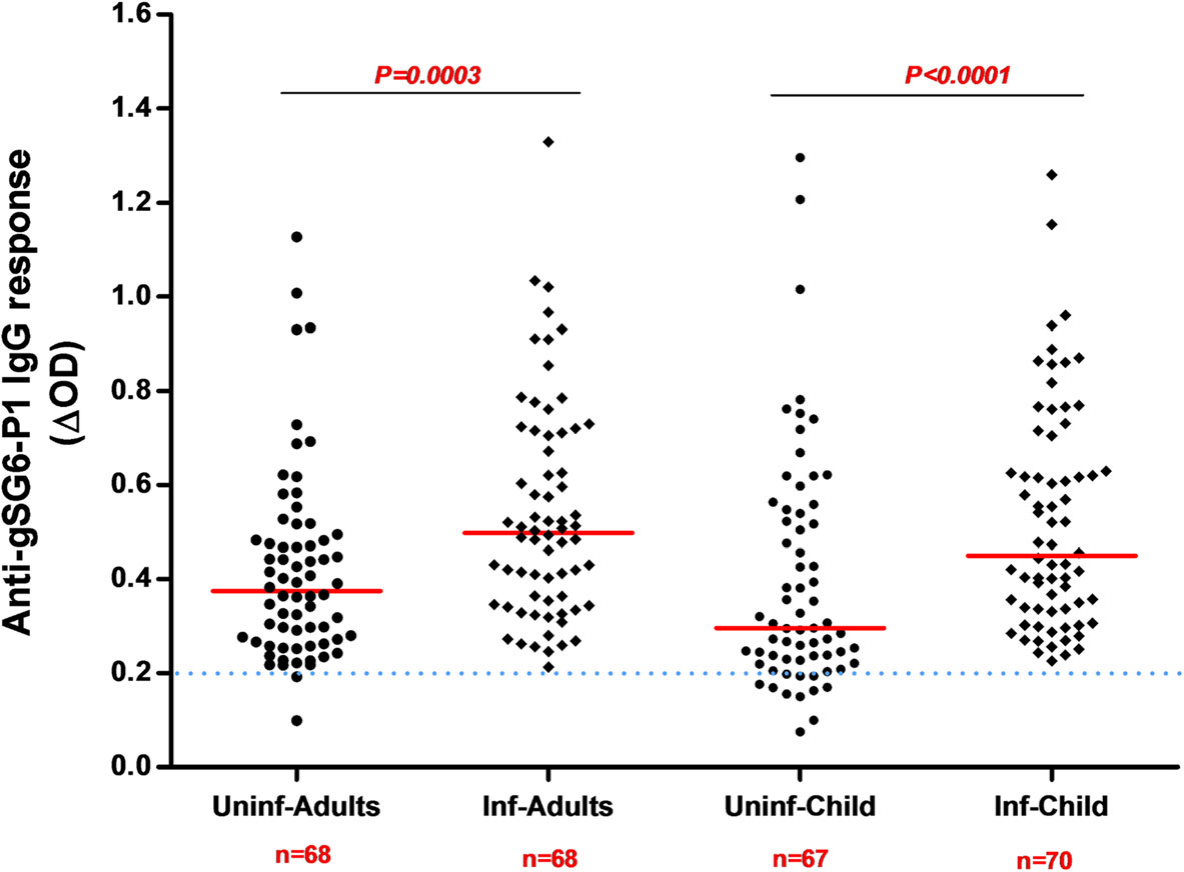
Anti-gSG6-P1 IgG levels according to the children and adults

Analysis according to the infection status (adults and children infected or not by malaria) indicated that the median anti-gSG6-P1 IgG level was significantly higher in infected than non-infected adults (Mann-Whitney U-test: *U *= 1488, *n*_1_ = 68, *n*_2_ = 68, *P* = 0.0003) and children (Mann-Whitney U-test, *U *= 1433, *n*_1_ = 67, *n*_2_ = 70, *P* < 0.0001) (Fig. 5).

### Entomological patterns of the study sites

In total, 4107 mosquitoes were caught in the four districts. The population was composed of 93.1% of *Culex* spp., 6.8% of *Anopheles* spp. and 0.2% of *Aedes* spp. (Table 4). The majority of *Anopheles* specimens were caught in the central districts of Tounouma and Dogona (259/278, 93.2%). CDC traps provided 72.3% (201/278) of the *Anopheles* samples and 86.1% (173/201) of them were collected with indoor CDC traps. Indoor resting females caught by spraying represented 27.7% (77/278) of the *Anopheles* samples.

**Table 4:** Mosquito species collected in the four districts with CDC traps and insecticide spraying

District	*An. gambiae* (*s.l.*)		*Aedes* spp.		*Culex* spp.		Total	
	*n*	%	*n*	%	*n*	%	*n*	%
Yeguere	11	6.0	1	0.5	170	93.4	182	4.4
Secteur 25	8	7.0	2	1.8	104	91.2	114	2.8
Tounouma	214	7.2	2	0.1	2760	92.7	2976	72.5
Dogona	45	5.4	2	0.2	788	94.4	835	20.3
Total	278	6.8	7	0.2	3822	93.1	4107	100

*Abbreviation*: *n*, total number of mosquitoes

PCR-based species identification of the *An. gambiae* (*s.l.*) females (*n* = 278) was successful for 273 specimens (four specimens from Tounouma and one form Dogona were not identified) (Table 5). The most frequently identified species was *An. arabiensis* (83.2%, 227/273), followed by *An. coluzzii* (10.6%, 29/273) and *An. gambiae* (6.2%, 17/273) (Table 3). *Anopheles gambiae* and *An. arabiensis* were present in all four districts, but with higher frequencies in the central ones (Tounouma and Dogona). *Anopheles arabiensis* represented 81.5% (185/227) of catches in Tounouma, whereas *An. gambiae* was predominant in Dogona (47.1%, 8/17) (Table 3). *Anopheles coluzzii* was only found in Tounouma and Dogona.

**Table 5:** Identification of malaria vector species in the four districts

District	*An. gambiae*			*An. coluzzii*			*An. arabiensis*			Total		
	*n*	%	Positive for *P.f*	*n*	%	Positive for *P.f*	*n*	%	Positive for *P.f*	*n*	%	Positive for *P.f*
Yeguere	1	9.1	0	0	0.0	0	10	90.9	0	11	4.0	0
Secteur 25	3	37.5	0	0	0.0	0	5	62.5	0	8	2.9	0
Tounouma	5	2.4	0	20	9.5	1	185	88.1	4	210	76.9	5
Dogona	8	18.2	0	9	20.5	0	27	61.4	0	44	16.1	0
Total	17	6.2	0	29	10.6	1	227	83.2	4	273	100	5

*Abbreviations*: *n*, total number of individuals per species (four specimens were not identified in Tounouma and one in Dogona); *P.f*, *Plasmodium falciparum*

Moreover, in these 273 *Anopheles* females, *P. falciparum* was found only in specimens collected in Tounouma, but not in Yeguere, Secteur 25 and Dogona. In addition, in Tounouma, only *An. coluzzii* (5.0%, 1/20) and *An. arabiensis* (2.2%, 4/185) were infected by *P. falciparum* (Table 5).

Analysis of the available blood-fed mosquitoes (*n* = 100) showed that in Tounouma, Dogona and Yeguere, ≥ 25% had a human blood meal and ≥ 15% a mixed meal (Table 6). Blood-fed females were not collected in Secteur 25. There was no difference in the blood meal origin distribution in the four districts (Kruskal-Wallis H-test, *χ*^2^ = 0.088, *df* = 2, *P* = 0.9565).

**Table 6:** Blood meal origin according to the district

District	Animals							Human		Mixed		Total
	Cattle	Sheep	Donkey	Pig	Dog	Other	%	*n*	%	*n*	%	*n*
Yeguere	3	0	2	0	1	0	54.5	3	27.3	2	18.2	11
Secteur 25	0	0	0	0	0	0	0	0	0	0	0	0
Tounouma	9	6	9	2	0	1	55.1	14	28.6	8	16.3	49
Dogona	1	9	7	3	3	1	60.0	10	25.0	6	15.0	40
Total	13	15	18	5	4	2	57.0	27	27.0	16	16.0	100

*Abbreviations*: *n*, number of blood-fed *Anopheles* females; Other, other animals not determined; Mixed, animal and human meal

## Discussion

The present study was carried out in four districts of Bobo-Dioulasso to evaluate the spatial disparities in malaria transmission to improve disease control in an urban setting. Despite the significant coverage of mosquito nets, the transmission of malaria occurred in the four districts [21]. Prevalence was higher in the central districts (Tounouma and Dogona) than in the peripheral ones, in relation with the presence of the Houet River. The human population exposure to *Anopheles* bites was confirmed by assessing the specific IgG response to the gSG6-P1 salivary peptide. Exposure was also higher in Tounouma and Dogona. Moreover, parasitaemia in children was higher in Bobo-Dioulasso compared to what was observed in Ouagadougou in 2004. The mean of the parasitaemia among positive children was 602 *P. falciparum* asexual parasites/μl of blood (95% CI: 545–665) [22]. Furthermore, prevalence was higher than the value reported by Yaméogo et al. [23] in 2012 for Bobo-Dioulasso that was 14.05%. This result could be explained by inadequate mosquito net use. Although people declared they slept under their bednet the night before the survey, we did not control this by house visiting at night or early in the morning. This can be considered as a limitation of the study. Recently, Taylor et al. [24] showed that ownership of mosquito nets increased in Burkina Faso from 6% in 2003 to 57% in 2010. Similarly, in Niger, ownership increased to 65.1% in all households and to 74.6% in households with children under 5; however, the authors found that only 33% of people had slept under their mosquito net the night before the survey [25]. On the other hand, a robust use of mosquito control tools (long lasting insecticide impregnated nets, mosquito coils, insecticide sprays) could lead to a change in the vector biting behaviour. This was observed in Benin, where 26.4% of all *An. funestus* specimens was caught after 06:00 h [26]. Thus, in Bobo-Dioulasso, transmission could also occur early in the morning when people leave their mosquito net. At the end, *An. arabiensis* was the main vector in Bobo-Dioulasso as previously reported by Dabiré et al. [13, 27]. The presence of *An. arabiensis* could also help understanding the persistence of malaria transmission. Indeed, its exophilic behaviour could reduce the effectiveness of vector control interventions that specifically target endophilic species, thus allowing residual transmission outside houses.

The higher malaria risk in central areas of Bobo-Dioulasso was relatively unexpected. Indeed, in Burkina-Faso, the risk of malaria is generally considered to be more elevated in suburban than central areas because of the persistency of rural features that favour the presence of malaria vectors [22]. The opposite situation in Bobo-Dioulasso could be linked to the specific environmental situation, characterized by an important hydrographic network within the town and urban agricultural activities that provide breeding sites for malaria vectors even if the river is polluted (organic pollutants or heavy metals and oil) [13, 28] (Fig. 5). Although larval prospections were not conducted in our study which can be considered as a limitation, presence of larvae in the Houet river was previously observed [13]. The presence of *Anopheles* larvae in polluted water was previously reported in Dakar, Accra, Dar es Salaam, and Lagos [6, 29–31]. In agreement, in Secteur 25 where the gSG6-P1 IgG response and malaria prevalence were the lowest, 69.3% of households were located more than 500 m from the hydrographic network. Conversely, in Tounouma and Dogona where the gSG6-P1 IgG responses and malaria prevalence were the highest, 73.1 and 63.8% of the households, respectively, were less than 200 m from the hydrographic network. Similar results were previously reported [15–17, 32].

## Conclusions

**Fig. 6:**
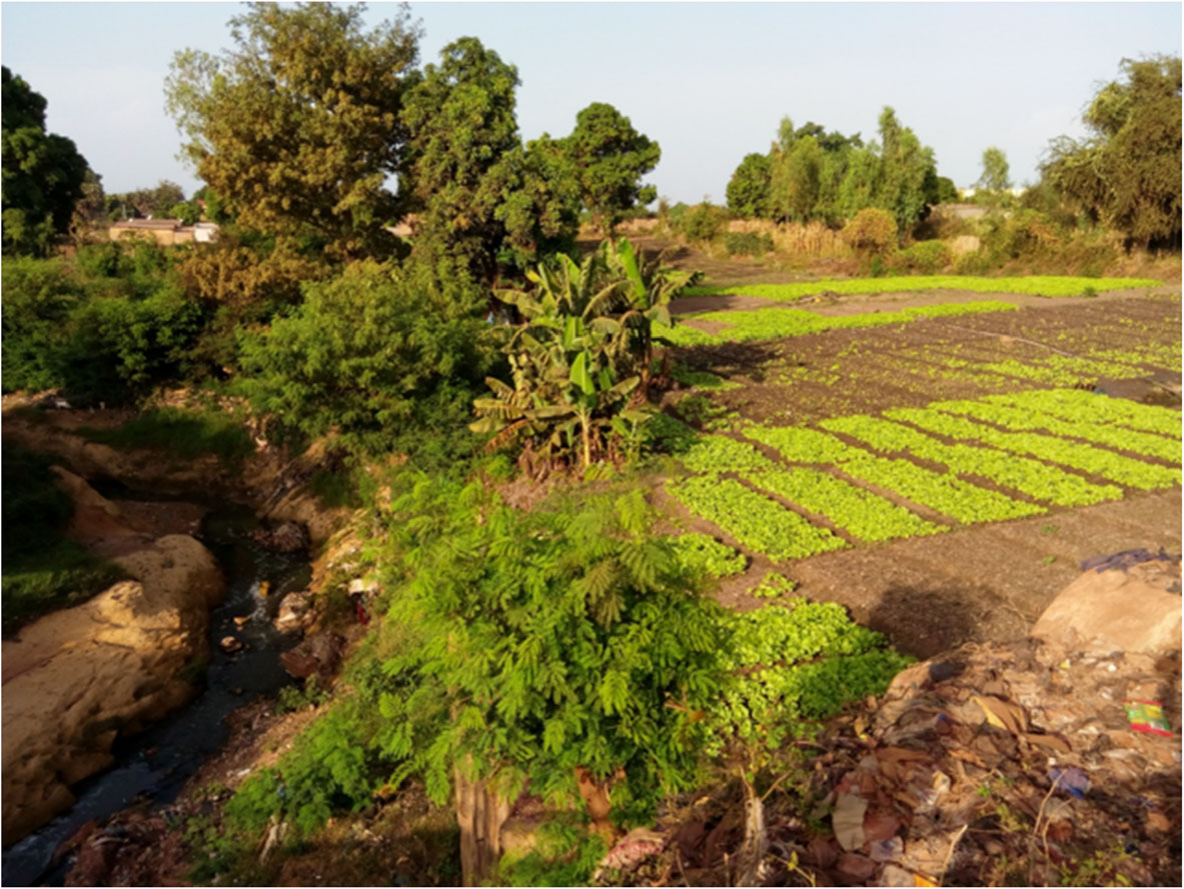
Urban agricultural activities in the central district of Dogona

The transmission of malaria was investigated in four districts of Bobo-Dioulasso that were selected to illustrate the great ecological diversity of the urban environment. District disparities in malaria prevalence were observed in children, highlighting that they were particularly exposed to malaria in the central part of the town. Environmental factors, such as urban agricultural activities along the Houet River favor the presence of malaria vectors and then host-vector contact (Fig. 6). Moreover, the major vector, *An. arabiensis*, is an exophilic species against which mosquito nets are less efficient. If sociological factors, such as a good education level, can strongly influence malaria impact within a population, environmental factors may be improved by better urban planning and management, calling for awareness to build sustainable healthy cities.

## Abbreviations

Ab: Antibody
ELISA: Enzyme-linked immunosorbent assay
Ig: Immunoglobulin
LLIN: Long-lasting insecticide impregnated nets
mRDT: Malaria rapid diagnostic test
OD: Optical density
PBS: Phosphate-buffered saline
PCR: Polymerase chain reaction
PNLP: Programme National de Lutte Contre le Paludisme (National Malaria Control Programme
WHO: World Health Organization
